# Increasing human monoclonal antibody cloning efficiency with a whole-cell modified immunoglobulin-capture assay (mICA)

**DOI:** 10.3389/fimmu.2023.1184510

**Published:** 2023-06-02

**Authors:** Sara Siris, Camilla A. Gladstone, Yanping Guo, Radhika Patel, Christopher L. Pinder, Robin J. Shattock, Paul F. McKay, Paul R. Langford, Fadil A. Bidmos

**Affiliations:** ^1^ Section of Paediatric Infectious Disease, Department of Infectious Disease, Imperial College London, London, United Kingdom; ^2^ Flow Cytometry Core Facility, National Heart and Lung Institute, Imperial College London, London, United Kingdom; ^3^ Section of Virology, Department of Infectious Disease, Imperial College London, London, United Kingdom

**Keywords:** *Neisseria meningitidis*, *Streptococcus pneumoniae*, reverse vaccinology 2.0, human monoclonal antibodies, vaccines, plasmablast enrichment, B-cell

## Abstract

Expression cloning of fully human monoclonal antibodies (hmAbs) is seeing powerful utility in the field of vaccinology, especially for elucidating vaccine-induced B-cell responses and novel vaccine candidate antigen discovery. Precision of the hmAb cloning process relies on efficient isolation of hmAb-producing plasmablasts of interest. Previously, a novel immunoglobulin-capture assay (ICA) was developed, using single protein vaccine antigens, to enhance the pathogen-specific hmAb cloning output. Here, we report a novel modification of this single-antigen ICA using formalin-treated, fluorescently stained whole cell suspensions of the human bacterial invasive pathogens, *Streptococcus pneumoniae* and *Neisseria meningitidis*. Sequestration of IgG secreted by individual vaccine antigen-specific plasmablasts was achieved by the formation of an anti-CD45-streptavidin and biotin anti-IgG scaffold. Suspensions containing heterologous pneumococcal and meningococcal strains were then used to enrich for polysaccharide- and protein antigen-specific plasmablasts, respectively, during single cell sorting. Following application of the modified whole-cell ICA (mICA), ~61% (19/31) of anti-pneumococcal polysaccharide hmAbs were cloned compared to 14% (8/59) obtained using standard (non-mICA) methods – representing a ~4.4-fold increase in hmAb cloning precision. A more modest ~1.7-fold difference was obtained for anti-meningococcal vaccine hmAb cloning; ~88% of hmAbs cloned *via* mICA versus ~53% cloned *via* the standard method were specific for a meningococcal surface protein. VDJ sequencing revealed that cloned hmAbs reflected an anamnestic response to both pneumococcal and meningococcal vaccines; diversification within hmAb clones occurred by positive selection for replacement mutations. Thus, we have shown successful utilization of whole bacterial cells in the ICA protocol enabling isolation of hmAbs targeting multiple disparate epitopes, thereby increasing the power of approaches such as reverse vaccinology 2.0 (RV 2.0) for bacterial vaccine antigen discovery.

## Introduction

The generation of human monoclonal antibodies (hmAbs) from B cells, facilitated by advanced high-throughput molecular tools, is enabling an increased understanding of the human immune response to both foreign and autoantigens. For example, advances in hmAb cloning are being exploited in detailed investigations into the immune response to disseminated infection, some of which are revealing the identities of candidate vaccine antigens. Reverse vaccinology 2.0 (RV 2.0) is one such sequential approach in which hmAb generation is followed by functional characterisation of pathogen-specific hmAbs and ultimately, structural analysis-based rational design of functional hmAb targets (1, 2).

Fluorescently tagged molecular probes targeting surface-expressed IgG have been employed in RV 2.0 studies to isolate high proportions of antigen-specific hmAbs from a heterogeneous pool of antibody-producing cells. These molecular probes have been used mainly for antibody-producing cells exhibiting cell surface presentation of the immunoglobulin (Ig) molecule, e.g., memory B-cells and plasma cells (3–6). Methods relying on cell-surface expression of Ig are, therefore, not applicable to plasmablasts, which are known to secrete Ig to the extracellular milieu instead of cell-surface presentation (7). These plasmablasts are very important to the pathogen-specific hmAb cloning process because a transient peak of this B-cell subpopulation at 6 – 16 days post-infection (8, 9) composes up to 80% of the total B-cell population (9), and is characterised by high numbers of cells actively differentiating and proliferating (Ki-67+; HLA-DR+) in response to acute infection or immunisation. Hence, a significant proportion of plasmablasts tend to be pathogen-specific, making this subpopulation an ideal target for hmAb cloning (10, 11). Within a hmAb-cloning context, this transient property is advantageous, thus fostering the exploitation of the Ig secretory capacity of plasmablasts in enrichment protocols.

A number of techniques exist for the detection and isolation of antigen-specific plasmablasts. For example, Clargo et al. reported a “fluorescent foci method” wherein the accumulation of secreted antibodies specific for, and in the immediate vicinity of, a solid-phase antigen was visualised with fluorescence microscopy before individual antibody-secreting cells were isolated using a micromanipulator device (12). Lin et al. described an *in vivo* protocol that involved intrasplenic transplantation of irradiated severe combined immunodeficient (SCID)/beige mice with human peripheral blood mononuclear cells (PBMCs) premixed with a target antigen. This *in vivo* technique resulted in the induction of specific human plasmablasts in the mouse recipients, equivalent to 18- to 20-fold enrichment (13). Other multiplex methods that permit high-throughput screening of secreted mAbs include: engraving of individual cells into 50 mm-diameter microwells produced *via* soft lithography on a poly(dimethylsiloxane) polymer (PDMS) template followed by screening of individual wells for secretion of antigen-specific mAbs (14), and nanoparticle-based microfluidic co-encapsulation (15). These protocols, while preserving the integrity of the RNA template for downstream hmAb cloning protocols, are complex techniques and require highly specialised equipment, which are ordinarily unavailable in many research laboratories.

Diffusion of secreted IgG away from the originating plasmablast can be inhibited using cell-surface affinity matrices. Pinder et al. described an IgG-capture assay (ICA) that utilised recombinant vaccine antigens to isolate specific plasmablasts from vaccinated individuals, enhancing the hmAb cloning efficiency by several orders of magnitude (from 1.37% without ICA to 94% with ICA) (16). So far, only single antigen probes have been employed with the ICA or other enrichment approaches, particularly in studies where the immune response to a target antigen is being investigated. However, for the discovery of novel bacterial vaccine candidates using RV 2.0, whole cells (containing the entire surface-expressed proteome of a target bacterial pathogen) would be ideal as probes, to capture the full breadth of the pathogen-specific plasmablast response. A suspension containing strains of disparate surface antigenic repertoires would allow coverage of the extensive antigenic variability exhibited by several bacterial pathogens. To our knowledge, no evidence for the use of whole cells in an enrichment protocol exists in published literature. We therefore hypothesised that the ICA could be adapted for use with whole bacterial cells for high enrichment of plasmablasts of interest. Here, we provide proof-of-principle for the isolation of pathogen-specific plasmablasts from frozen PBMCs obtained from healthy recipients of pneumococcal polysaccharide and meningococcal protein vaccines. Using a modified ICA protocol, we demonstrate that a cocktail of heterologous strains can be used to isolate both clonal and sequence-specific plasmablast-derived hmAbs targeting individual surface-exposed epitopes. Our results further demonstrate the application of the ICA to the search for novel bacterial vaccine targets, especially from convalescing patients where challenges relating to volume (paediatric patients, for example) and timing of blood sampling require enrichment.

## Materials and methods

### Isolation of PBMCs

Blood was collected pre-vaccination and 7 days post-vaccination from three healthy adult (age range 25 – 35 years old) recipients of pneumococcal polysaccharide only (PPSV23), pneumococcal conjugate (PCV13) and meningococcal protein (4CMenB) vaccines, with informed consent. Volunteers were laboratory staff working directly on projects involving live cultures of both meningococcal and pneumococcal strains and received the vaccines as part of occupational health policy. Blood sampling from these volunteers for this present study was, therefore, opportunistic. PBMCs were extracted using the density-gradient centrifugation methods described in the Leucosep^®^ instruction manual (Greiner Bio-One; Item no.: 227290) and stored at -80°C in freezing medium (90% fetal bovine serum, FBS; 10% dimethyl sulfoxide, DMSO).

### Bacterial strains, purified polysaccharides and protein antigens

Purified pneumococcal polysaccharides (PPS) of serotypes 6A, 6B, 7F and 14 were obtained from the American Type Culture Collection (ATCC). Following reconstitution in pyrogen-free distilled water at a 5 mg/ml concentration, PPS were stored at -80°C. Bacteria (see **Table 1** for details and sources) were routinely cultured as follows: Tryptic Soy broth (TSB) supplemented with 0.5% Yeast Extract in cell culture flasks with vented caps for pneumococci; and Oxoid Chocolate GC selective agar (Thermo Fisher Scientific) for meningococci. Cells were harvested from media, washed thrice in sterile phosphate buffered saline (PBS) before fixing in 0.5% v/v formalin at room temperature for 4 hours. Fixed cells were washed again in PBS before resuspension to an optical density at 600 nm (OD600) of ~0.5. *Escherichia coli* strain HST08 (Stellar™ competent cells, Takara Bio) harbouring the ampicillin-resistant (Amp^r^) AbVec plasmids (gift from Hedda Wardemann; Addgene plasmid #80795; http://n2t.net/addgene:80795; RRID: Addgene_80795) (17) was grown in terrific broth/agar (Thermo Fisher Scientific) supplemented with ampicillin (100 μg/ml w/v). *Actinobacillus pleuropneumoniae* strain MIDG2331 (18) was cultured in Brain Heart Infusion broth/agar (BHI, Oxoid) supplemented with 0.01% nicotinamide adenine dinucleotide (NAD). All bacterial strains (see **Table 1**) were cultured under aerobic conditions at 37°C, 5% CO_2_ for 16 – 18 hours.

**Table 1:** Bacterial strains employed in this study.

Species	Strain/Isolate alias	Serotype/Serogroup	Sequence Type (Clonal Complex)
*Escherichia coli*	BL21	–	–
	HST08	–	–
*Streptococcus pneumoniae*	D39^a^	2	595
	H155340798^b^	4	206
	IOKOR1373-9^c^	6A	490
	M225-6B^c^	6B	90
	H152940600^b^	7F	191
	H133120215^b^	8	53
	M117-14^c^	14	162
	BHN 100^c^	19F	236
	H134020907^b^	22F	433
	OXC-1417-23F^c^	23F	36
	SM-P03	33F	- ^ND^
*Neisseria meningitidis*	MC58	B	74 (CC 32)
	NZ98/254	B	42 (CC 41/44)
	M07-240669^e^	B	1097 (CC 41/44)
	M10-240474^e^	B	269 (CC 269)
	M10-240480^e^	B	1194 (CC 41/44)
	M11-240123^e^	B	3537 (CC 11)
	M11-240016^e^	B	1096 (CC 32)
	M14 240312^d^	B	2922 (CC 41/44)
*Actinobacillus pleuropneumoniae*	MIDG2331^f^	–	–

^ND^No data.

a Obtained from American Type Culture Collection (ATCC).

b Obtained from David Litt, UK Health Security Agency (UKHSA).

c Obtained from Jeremy Brown, University College London, UK.

d Isolate from patient recruited into another study conducted by FB and PL.

e Obtained from Meningococcal Reference Unit, UKHSA.

f Susanna Williamson, Animal and Plant Health Agency (APHA), UK.

### Indirect enzyme-linked immunosorbent assay

Bacterial cells or PPS suspensions in Carbonate-Bicarbonate buffer (Sigma-Aldrich, cat. no. C3041) were used to coat wells of polystyrene 96-well plates for 16 hours at 4°C. All subsequent steps were performed at room temperature. Wells were washed thrice with assay buffer (PBS-T: PBS, 0.05% Tween-20) to remove excess unattached cells or PPS. Non-specific binding of antibodies was prevented in an initial blocking step for 1 hour using PBS-T supplemented with 3% w/v bovine serum albumin (BSA). Appropriate dilutions of plasma or hmAbs in PBS-T were added and binding allowed to proceed for 1 hour before removal of unbound antibodies with three PBS-T washes. An anti-human IgG alkaline phosphatase conjugate (Sigma-Aldrich, cat. no. A9544) at a 1:2000 dilution in PBS-T was added to wells for 1 hour. Following five washes with PBS-T, colour development was induced *via* the addition of a phosphatase substrate (Bio-Rad, cat. no. 172-1063) as per manufacturer’s protocols. Signal intensity was measured at OD405 nm using a Versamax microplate reader (Molecular Devices).

### Modified IgG capture assay

Strains IOKOR1373-9 (6A), H160120907(7F), M117-14 (14) and BHN 100 (19F) were selected for the PCV13 ICA based on immunogenicity profiles obtained following vaccination, as reported in (19). All four strains composed a single suspension in an equal amount (all individually normalised to an OD600 ~0.5 before equal amounts added together for the suspension). For the PPSV23 mICA, strains D39 (serotype 2), H133120215 (serotype 8), H134020907 (serotype 22F) and SM-P03 (serotype 33F) in equal amounts were used as the probe (PPSV23 suspension). The meningococcal mICA involved three different suspensions of meningococcal serogroup B strains: (3x) MC58, NZ98/254 and M10-240480; (5x) 3x strains plus M07-240669 and M11-240016; and (7x) 5x strains plus M10-240474 and M11-240123. The mICA was performed as comprehensively described in (20). Briefly, formalin-fixed cells were labelled with 100 μg/ml of 5-Carboxyfluorescein Succinimidyl Ester (5-FAM SE, Sigma-Aldrich cat. no. 21878) as previously described (21), to enable the gating strategy described hereafter. An IgG affinity matrix was assembled on plasmablasts using anti-CD45 streptavidin and biotin anti-human IgG. Following secretion and capture of IgG, labelled bacteria (FAM SE) were mixed with plasmablasts in assay buffer (PBS, 1% w/v BSA) and incubated at room temperature in the dark for 20 minutes (**Figure 6 of Supplementary Information**). Plasmablast-bacteria complexes were washed once, prior to staining of plasmablasts with the following antibodies: anti-CD3 APC (BioLegend, Clone HIT3a), anti-CD14 APC (BioLegend, Clone M5E2), anti-CD19 Brilliant Violet 421 (BioLegend, Clone HIB19), anti-CD20 Brilliant Violet 605 (BioLegend, Clone 2H7), anti-CD27 PE (BioLegend, Clone M-T271), anti-CD38 PerCP/Cy5.5 (BioLegend, Clone HIT2), anti-IgM Brilliant Violet 650 (BioLegend, Clone MHM-88), anti-IgD Brilliant Violet 786 (BD Biosciences, Clone IA6-2), and anti-IgA Alexa Fluor 594 (Jackson Immunoresearch, 109-585-011-JIR). PBMC Fluorescence-Minus-One (FMO) controls were included in these experiments. Cellular complexes were washed once before analysis using a BD FACSAria™ III (BD Biosciences). Pathogen-specific plasmablasts were gated, as follows: CD3/14-, CD20-, CD19+, CD27^hi^, CD38^hi^, IgA-, IgD-, IgM-, FAM SE+. Cells were individually sorted into wells of 96-well plates containing 10 μl of catch buffer (10 mM Tris pH 8.0, 10 units RNAsin). Plates were stored at -80^0^C until further processing.

### Cloning, sequencing and expression of hmAbs

Cloning of the variable regions of IgG heavy and light chains (V_H_; and VL = κ/λ) into relevant AbVec IgG expression vectors and subsequent expression of hmAbs was performed as previously described (22). A slight modification to the protocol involved substituting restriction endonuclease cloning with a ligation-independent method using the InFusion HD Cloning Plus system (Takara Bio) for enhanced cloning efficiency. The sequences of all V gene fragments were obtained by Sanger sequencing and analysed using the IGBLAST tool hosted on the National Center for Biotechnology Information website (www.ncbi.nlm.nih.gov/igblast/). Calculations of the replacement-to-silent mutation ratios (*d_N_*/*d_S_*) were performed using approximate methods (23). Thresholds of >2.9 and >1.5 are indicative of positive antigen-driven selection for replacement mutations for CDR and FR regions, respectively (24).

### Flow cytometry

A high-throughput multi-well plate format was used to assess reactivity of hmAbs in flow cytometry experiments. In each well, 60 μl of bacterial suspensions normalised to OD 600 of ~0.5 were mixed with 1 μg/ml of hmAb and incubated for 45 minutes at room temperature. Unbound hmAb was removed by one wash with PBS (3000 x g, 5 minutes). 5-FAM SE-stained bacteria were resuspended in 5 μg/ml of the secondary antibody, Alexa Fluor^®^ 488 Goat Anti-Human IgG (H+L) (Life Technologies™), and the suspension incubated for 30 minutes at room temperature. Subsequently, cells were washed to remove unbound antibodies before resuspending in PBS. Fluorescence intensities were measured and analysed using a BD LSRFortessa™ Cell Analyzer (BD Biosciences) and FlowJo software version 10. Heat maps were generated using GraphPad Prism v9.4.1.

### SDS-PAGE and western blotting

Protein epitope-specific hmAbs were assayed *via* western blotting, as previously described (22). Briefly, meningococci were lysed in 1x Laemmli buffer at 98^0^C, 5 minutes. Proteins in the lysates were electrophoresed using 10% Bis-Tris Protein Gels at 70 V for 5 hours. Following electrophoresis, proteins were transferred onto PVDF membranes. Non-specific binding was inhibited by blocking membranes with PBS-T plus 5% skimmed milk before probing with 0.5 μg/mL of hmAbs. After washing with PBS-T (thrice, 5 minutes each), membranes were incubated with a 1:2000 dilution of anti-human IgG peroxidase conjugate (Sigma Aldrich, cat. no. A4416). Membranes were washed again to remove unbound antibodies and subsequently developed using the Azure Biosystems c600 imaging system.

### Sodium periodate assay

The polysaccharide epitope specificity of pneumococcal capsular hmAbs was further confirmed using the sodium periodate assay, as previously described (25). Microtitre wells of a 96-well plate coated with pneumococcal cells were rinsed with 50 mM sodium acetate before addition of 10 mM sodium periodate. Following incubation in the dark for 1 hour at room temperature, wells were rinsed again with 50 mM sodium acetate. Next, 50 mM sodium borohydride was added to each well and the plate incubated for 30 minutes at 23°C. After five washes with PBS-T, appropriate dilutions of hmAbs were added and the plate was incubated for another hour at room temperature. The plate was washed five times with PBS-T before addition of a 1:2000 dilution of goat anti-human IgG alkaline-phosphatase conjugate (Sigma Aldrich, cat. no. A9544). After 1 hour incubation at room temperature, the plate was washed again 5 times with PBS-T before colour development was affected using a phosphatase substrate. Signal intensity was obtained and analysed, as mentioned previously.

### Opsonophagocytic killing assay

Neutrophils were purified from freshly-obtained blood of healthy human donors using the MACSxpress magnetic purification system (Miltenyi Biotechnology, cat. No. 130-104-434), as per manufacturer’s instructions. Viable cells of pneumococcal strain M117-14 were pre-mixed with a capsule-specific hmAb (0.4 μg/mL) and 10% v/v baby rabbit complement (Bio-Rad), and the mixture incubated for 30 minutes at 37°C, with shaking at 150 rpm. Approximately 4 x 10^3^ colony-forming units (CFU) from this mixture were then transferred to wells of a clean microtitre plate. To each well containing the pneumococci-hmAb mixture, 4 x 10^5^ neutrophils were added (MOI of 1:100). The plate was subsequently incubated for 45 minutes at 37°C, with shaking at 150 rpm. The numbers of surviving bacteria were calculated by plating serial dilutions, and the results were expressed as a percentage of the inoculum CFU.

### Serum bactericidal assay (SBA)

Functional activity of anti-meningococcal hmAbs were assessed in SBAs, as previously described in (22). Briefly, 1 x 10^3^ CFU of viable meningococci were mixed with 4 μg of hmAbs and incubated at 37°C, 5% CO_2_ for 5 minutes. Exogenous human complement (lacking background bactericidal activity against meningococci) were added at 25% v/v and the incubation continued for 60 minutes. Meningococcal CFU was enumerated by plating samples on GC selective agar plates. A reduction in CFU by more than 50% of the inoculum was indicative of serum bactericidal activity.

## Results

### Vaccine-induced plasmablast expansion translates to enhanced functional antibody titres

**Figure 1:**
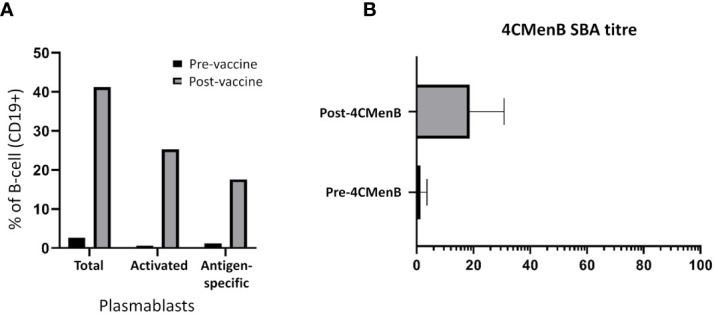
Analyses of the adaptive immune responses pre- versus post-vaccination. **(A)** Quantitative analysis of different subsets of the CD19+ B-cell population. The geometric mean of the sub-populations from PPSV23, PCV13 and 4CMenB vaccinees are represented in the figure as a fraction (in percentage, %) of the CD19+ B-cell population. **(B)** Titres of functional plasma-derived antibodies pre- and post-4CMenB vaccination.

An enhanced presence of circulating plasmablasts was apparent following staining of different subpopulations in FACS analyses. Plasmablasts represented 1.4% of the CD19+ B-cell population prior to vaccination in the three healthy subjects (range = 1.0 to 2.7%); 0.4% of these plasmablasts were discerned to be antigen-specific (“positive” for FAM-SE because of binding to stained bacteria). A ~12.5-fold increase in this subpopulation was observed in post-vaccination PBMCs, with plasmablasts comprising 7.3%, 9.9% and 75.1% of the CD19+ population following PPSV23, 4CMenB and PCV13 administration, respectively. Antigen-specific plasmablasts were also more prominent in post-vaccination PBMCs (average of 14.0%; range 10.5% – 24.6%); a significantly lower percentage of stained bacteria binding CD19+ cells was detected in the circulation of individuals pre-vaccination (**Figure 1A**). Enhanced serum bactericidal activity against three meningococcal strains, MC58, M10-240480 and NZ98/254 (matched for 4CMenB factor H binding protein (FHbp) and neisserial heparin binding antigen (NHBA) variants, and the outer membrane vesicle (OMV) component, respectively), was also discerned following 4CMenB vaccination indicating that vaccine-induced plasmablast proliferation translated into an increase in functional antibody titres in plasma (**Figure 1B**).

### Enhanced cloning of pathogen-specific hmAbs with mICA

**Figure 2:**
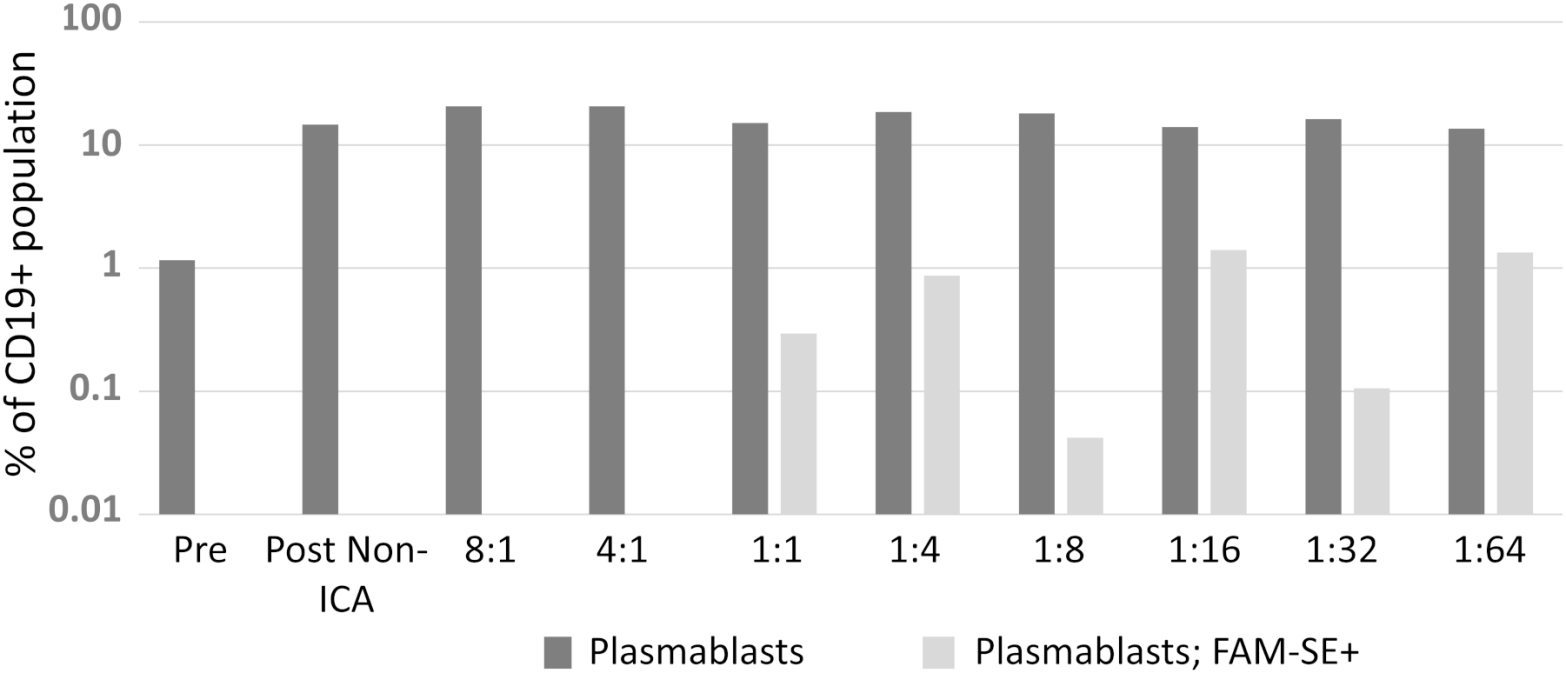
A suitable PBMC-to-bacteria ratio was first determined. Results from two biological replicates were comparable; hence data presented here is from one independent experiment.

Initially, the optimum ratio of PBMC-to-bacteria optimum for signal detection (for both cell types) during FACS was investigated by comparing eight ratios ranging from 8:1 through 1:1 to 1:64 ratios. Bacteria-binding CD19+ cells were identified in ratios 1:1 to 1:64, the 1:16 and 1:64 ratios performed best yielding identification of 1.4% and 1.3% of antigen-specific plasmablasts respectively (**Figure 2**).

**Figure 3:**
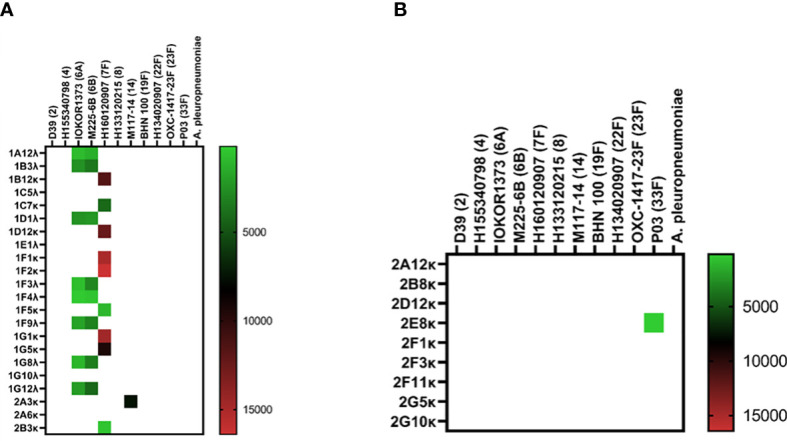
Reactivity of recombinant anti-pneumococcal mICA hmAbs in immunoassays. Reactivity of hmAbs cloned from PCV13 **(A)** and PPSV23 **(B)** vaccinees versus whole pneumococcal cells was assessed in flow cytometry experiments. Geometric mean fluorescent intensity (gMFI) from 10000 events are presented in the heat map.

Isolation of pneumococcus-specific hmAbs was first pursued with the 1:16 PBMC-to-bacteria ratio and using four pneumococcal strains as described in Methods; for the PCV13 mICA, 18 out of 22 recombinant hmAbs produced in HEK-293 cells were reactive in varying intensities with three of the four PCV13 serotypes used for enrichment (6A, 7F and 14; whole cells and PPS). No antibodies targeting the 19F capsule were isolated. Nine hmAbs bound to serotype 7F strain H152940600 while one hmAb (2A3κ) bound to strain serotype 14 strain M117-14. All eight hmAbs that bound serotype 6A strain IOKOR1373-9 were also reactive with a serotype 6B strain, M225-6B. A hmAb that contained a non-productive out-of-frame Vκ region, 1F5, was specifically reactive with strain H152940600; albeit, at levels lower than the remaining anti-7F hmAbs that possessed both heavy and light chains. With the exception of the 6A/6B cross-reactivity, none of the hmAbs exhibited cross-reactivity with strains expressing heterologous pneumococcal polysaccharides strongly suggesting that the targets of these hmAbs were non-protein, polysaccharide epitopes (**Figure 3A**). The specificity for the pneumococcal polysaccharide for a subset of these hmAbs was confirmed in ELISAs with purified polysaccharides as antigen; all five selected hmAbs including 2A3κ reacted specifically with their cognate polysaccharides (**Supplementary Information, Figure 1A**). This data was further supported by a sodium periodate assay that showed loss of interaction between 2A3κ and its cognate polysaccharide epitope. No similar loss of interaction between a meningococcal protein epitope-specific hmAb P09-2F7 and its cognate target was observed (**Supplementary Information, Figure 1B**). Considerably less success was obtained with the PPSV23 mICA as only one out of nine hmAbs was pneumococcus-specific (anti-33F polysaccharide; **Figure 3B**). Like the PCV13 mICA hmAbs, this sole PPSV23 anti-33F hmAb was non-reactive with heterologous pneumococcal serotypes.

**Figure 4:**
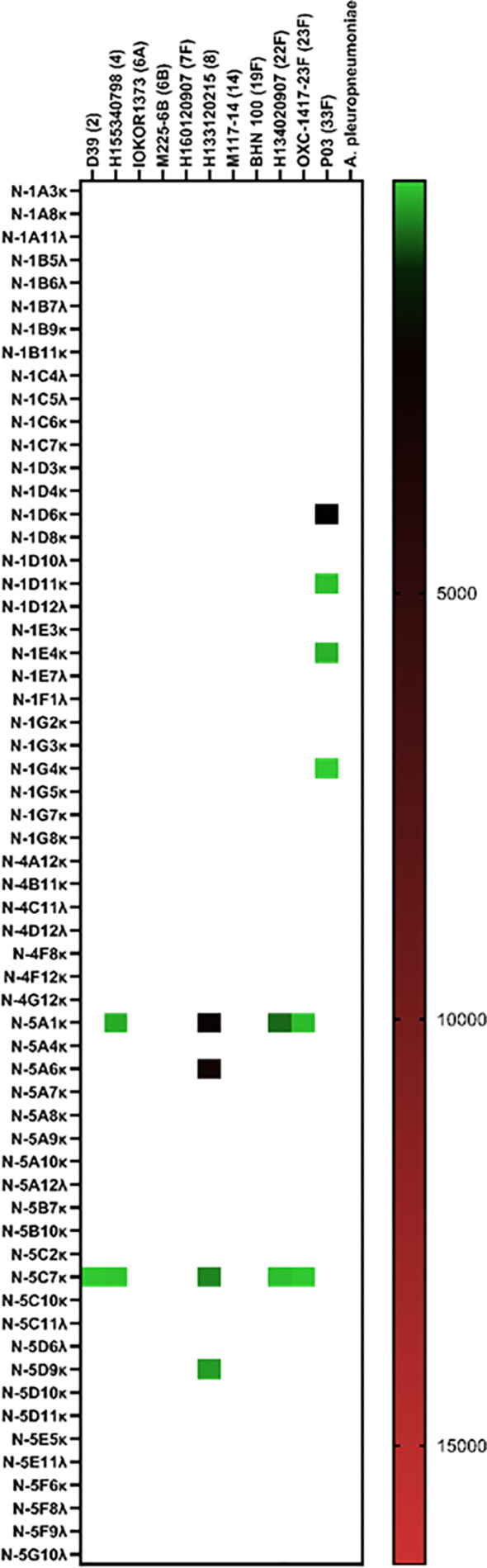
Cloning of anti-pneumococcal vaccine-induced hmAbs using standard methods. Geometric mean fluorescent intensity (gMFI) from 10000 events are presented in the heat map.

Without employment of the mICA, eight out of 59 hmAbs (13.6%) cloned from non-enriched plasmablasts bound to a pneumococcal surface antigen, four of which bound to the serotype 33F strain SM-P03 while another two bound to serotype 8 strain H133120215. Interestingly, the two-remainder hmAbs exhibited pneumococcal antigen specificity indicative of a non-polysaccharide cognate target as both showed significant cross-serotype reactivity (**Figure 4**).

**Figure 5:**
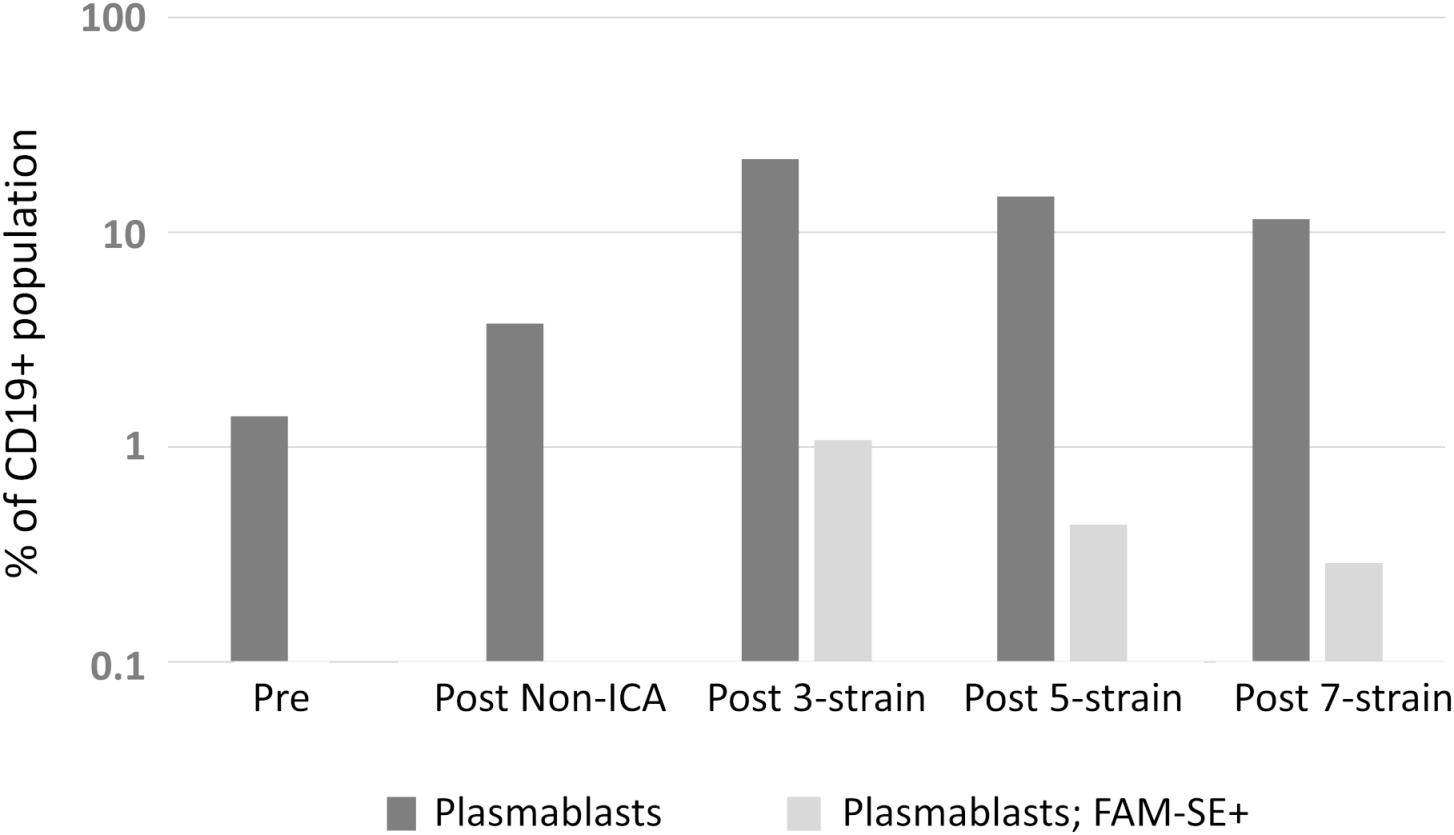
Assessment of the complexity limit of the antigen probe for mICA. Suspensions with 3-, 5- and 7-strain compositions were employed as probe and comparisons of level of enrichment were made using FACS.

Following from results of the anti-pneumococcal mICA, specifically the PCV13 mICA where no serotype 19F hmAbs were cloned, we sought to determine if a technical anomaly could account for the non-enrichment of serotype-19F plasmablasts, prior to executing the meningococcal mICA. To this end, we explored the complexity limit of the antigen probe, utilising three, five or seven heterologous meningococcal strains in a comparison assay. Precursory data from the single experiment conducted (sample availability was a limiting factor) suggested superior enrichment with the 3-strain suspension, with an inverse relationship between enrichment and complexity of the antigen pool (**Figure 5**).

**Figure 6:**
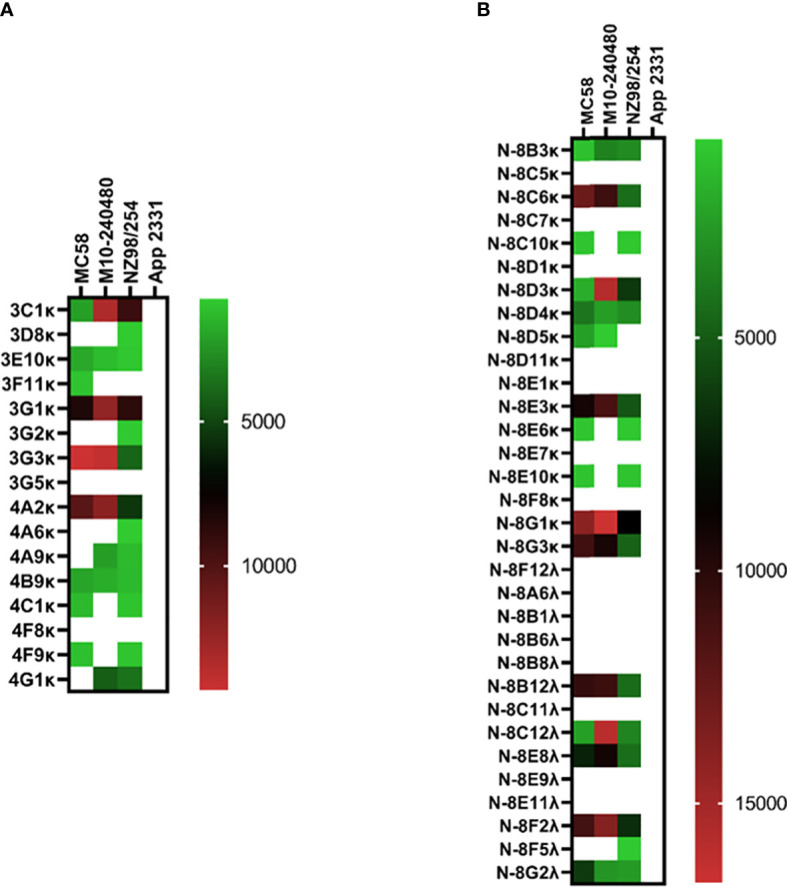
Reactivity of recombinant anti-meningococcal hmAbs in immunoassays. Flow cytometry assays were employed in the determination of mICA **(A)** and standard method **(B)** hmAb specificity for meningococcal surface epitopes. Data from these experiments were extracted as previously described herein. Geometric mean fluorescent intensity (gMFI) from 10000 events are presented in the heat map.

Like the pneumococcal mICA, enrichment with a meningococcal suspension (3-strain) enhanced precision of pathogen-specific hmAb cloning albeit by a modest degree; 14 out of 16 hmAbs (87.5%; **Figure 6A**) bound to a cognate epitope on the meningococcal surface compared to 53% without enrichment (17/32; **Figure 6B**). Western blotting following denaturing SDS-PAGE revealed that the targets of some of these vaccinee-derived anti-meningococcal hmAbs were highly-specific linear protein epitopes and that these targets included composite proteins of the OMV component of 4CMenB (**Supplementary Information, Figure 2**).

Overall, of the 47 hmAbs cloned in this study with enrichment, 33 were antigen-specific (70.2%) representing a 2.6-fold higher cloning efficiency than the 27.5% (25 out of 91 hmAbs total) obtained with standard methods.

### Functional properties of cloned mICA hmAbs

We next assessed the functional property of cloned mICA hmAbs in opsonophagocytic killing (anti-pneumococcal) and serum bactericidal (anti-meningococcal) assays. Anti-pneumococcal hmAb 2A3κ mediated complement-dependent opsonophagocytosis of the homologous serotype 14 strain at a level comparable to the human PCV13-immune serum (94.0% reduction versus 98.5%, respectively; **Supplementary Information, Figure 3**). Conversely, none of the eight anti-meningococcal vaccinee-derived hmAbs assessed in SBAs possessed a bactericidal property. While three patient-derived anti-meningococcal hmAbs with known SBA property mediated complement-dependent killing of meningococci at different amounts (ranging from 1 to 10 μg), increases in CFU were observed for all vaccinee-derived meningococcal hmAbs following a 60-minute incubation in the presence of exogenous human complement (data not shown).

### Heterogeneity of vaccine antigen-specific hmAbs driven by somatic hypermutation

**Figure 7:**
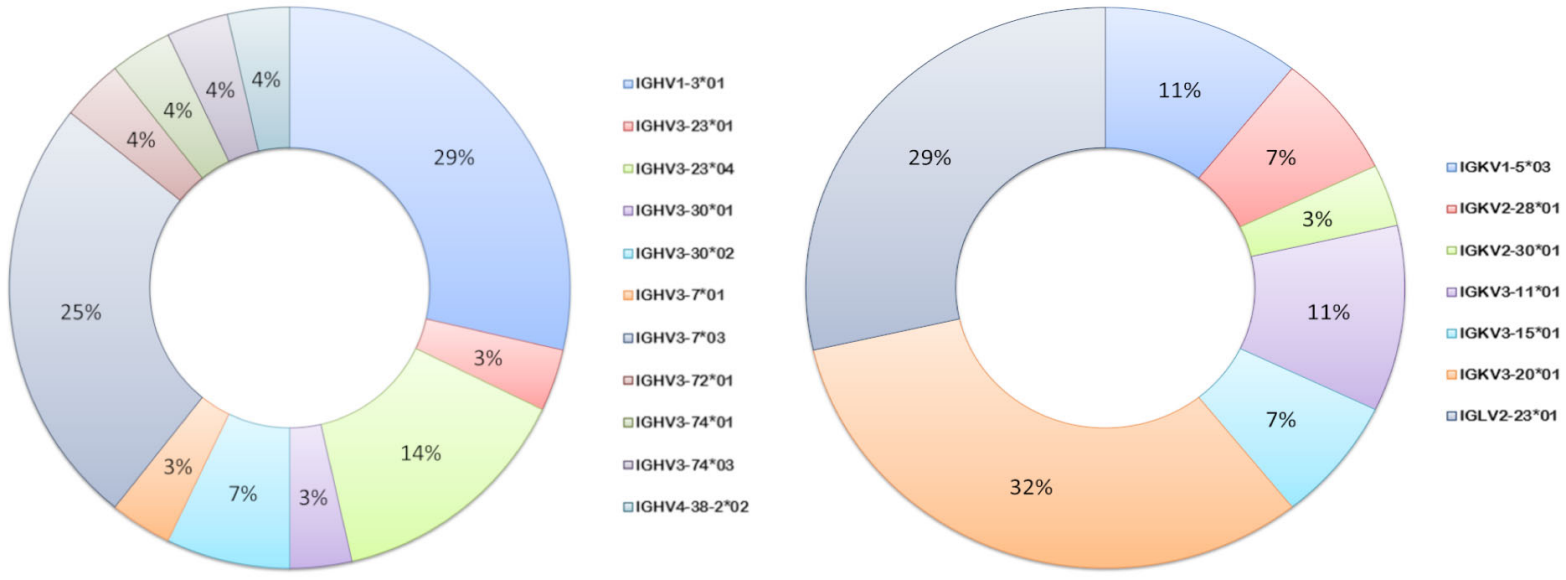
Ig V gene sequence usage in pneumococcal vaccine antigen-specific hmAbs. Frequencies of usage of shown Ig gene sequence families for heavy (left donut) and light (right donut) chains of pneumococcal polysaccharide-specific hmAbs.

Germline usage in the anti-pneumococcal hmAb panel tended towards serotype dependence and V gene sequence clonality; all eight anti-6A/6B hmAbs were composed of the IGHV1-3:IGλV2-23 pairing, while the majority of serotype 7F hmAbs (7 out of 9; 78%) possessed an IGHV3-7:IGκV3-20 pairing. Clonality was also maintained in the diversity gene (D) regions of the anti-6A/6B hmAbs (IGHD5-12*01) but not the anti-7F hmAbs (**Table 2**). The sole serotype 14 hmAb (2A3κ) possessed an IGHV4-38:IGκV3-15 pairing. The anti-33F hmAb group was an exception; two hmAbs possessed IGHV3-23:IGκV3-11, while the other two (N-1D11κ and N-1G4κ) possessed IGHV3-30:IGκV3-15 and IGHV3-30:IGκV3-20, respectively. With the exception of hmAbs 1F5 (anti-7F) and 2A3κ (anti-14) that belonged to the IGHJ3 family, all other anti-pneumococcal hmAbs possessed sequences in the IGHJ4 family (**Figure 7**).

**Table 2:** Sequence characterisation of the V gene segments (heavy and light chains) of antigen-specific hmAbs.

HmAb	VH family	HCDR sequence	HCDR3 length	DH family	JH family	VL family	LCDR3 sequence	LCDR3 length	JL family	R/S ratio
1A12λ	1-3	AREGGYTGCDLDY	13	5-12	4	2-23	CSYANSNPYD	10	1	2.1
1B3λ	1-3	AREGGYSGCDLDY	13	5-12	4	2-23	CSYANSNPYD	10	1	2.4
1B12κ	3-7	AREGAGGFDY	10	3-16	4	3-20	QEYGTSLKT	9	1	2.0
1C7κ	3-74	ARDSPTDLTLDV	12	2-21	4	2-28	MQALQTPWT	9	1	3.1
1D1λ	1-3	AREGVYSGCDLDY	13	5-12	4	2-23	CSYANSNPYD	10	1	3.2
1D12κ	3-7	AREGAGGFDY	10	3-10	4	3-20	QQYGTSPKT	9	1	4.2
1F1κ	3-7	AREGAGGFDY	10	3-16	4	3-20	QEYGTSLKT	9	1	3.3
1F2κ	3-7	AREGAGGFDY	10	3-10	4	3-20	QQYGTSPKT	9	1	4.2
1F3λ	1-3	AREGGYSGCDLDY	13	5-12	4	2-23	CSYANGNPYD	10	1	1.8
1F4λ	1-3	AREGAHSGCDLDY	13	5-12	4	2-23	CSYAAGNAYD	10	1	2.6
1F5κ	3-72	ARWLVVGETTSNPYDA	16	1-26	3	ND	ND	ND	1	ND
1F9λ	1-3	AREGGYSGCDLDY	13	5-12	4	2-23	CSYANGNAYD	10	1	2.7
1G1κ	3-7	AREGAGGFDY	10	3-16	4	3-20	QEYGTSLKT	9	1	2.8
1G5κ	3-7	AREGAGGFDY	10	3-10	4	3-20	QQYGSSPKT	9	1	2.0
1G8λ	1-3	AREGTYSGCDLDY	13	5-12	4	2-23	CSYANSNPYD	10	1	2.4
1G12λ	1-3	AREGAYSGCDLDY	13	5-12	4	2-23	CSYANSNPYD	10	1	4.2
2A3κ	4-38	ARMNAFDF	8	ND	3	3-15	QHYNAWPLT	9	4	1.8
2B3κ	3-7	AREGAGGFDY	10	3-16	4	3-20	QEYGTSLKT	9	1	2.7
2E8κ	3-23	ARSPGRPGTPGFWFDS	16	6-6	5	3-11	QQRSNWPLS	9	4	3.1
N-1D6κ	3-23	ARSPGITGTNGYWFDS	16	1-7	5	3-11	QQRSNWPLT	9	4	4.8
N-1D11κ	3-30	ATWGGYGGYDSSDYCVFDY	19	3-22	4	3-15	QQYSNWPPA	9	5	2.5
N-1E4κ	3-23	ANSPGKDGTPGFWFDP	16	1-7	5	3-11	QQRSNWPLS	9	4	3.2
N-1G4κ	3-30	AFWNGVDYNTKWLGGPFDF	19	2-21	6	3-20	QHYGETRRFT	10	3	3.5
N-5A1κ	3-23	AKVSWSRDTWDQDY	14	2-21	4	1-5	QQYASYPWT	9	1	3.1
N-5A6κ	3-7	ARGVWWAG	8	1-26	4	2-30	MQGAHWPYT	9	2	1.9
N-5C7κ	3-23	AKVSWSRENWDRDY	14	2-21	4	1-5	QQYAAYPWT	9	1	2.6
N-5D9κ	3-74	TRENFWSGWN	10	3-3	4	3-20	QSYGSSWT	8	1	4.0
3C1κ	4-34	VRGRRRIPPLASPRIQSQRRFYMDV	25	2-2	6	3-20	QQYGTSFWT	9	1	4.3
3E10κ	4-34	ARGRRAGYRGERNFFAPMVAAHYFDS	26	2-15	4	3-20	QQYATSPET	9	2	3.6
3F11κ	4-34	ARGRRAGFRGERNFIAAVVAAHYFDY	26	2-15	4	3-20	QQYGSSPET	9	2	1.8
3G1κ	4-34	ARARAVLTAFGSPRRERKAGERRNWFDP	28	3-3	5	3-20	QQYGSSPYT	9	2	2.4
3G2κ	4-34	ARGRRAGFRGERNFIAAVVASHYFDS	26	2-21	4	3-20	QQYGSSPET	9	2	1.9
3G3κ	4-34	SRGRRAGFRGERNFFAAMVASHYFDS	26	5-18	4	3-20	QQYETSPET	9	2	2.3
3G5κ	4-34	ARGRLIGGPFRQEPKSRQRSNWFDP	25	3-10	5	3-15	SNMMAGLRGR	10	1	3.6
4A2κ	4-34	ARGRLVRSPFGHQRVPRQKLNWFDP	25	6-13	5	3-15	QQYNNRPPWT	10	1	2.0
4A6κ	3-30	AKDVWGSYRPYYFDY	15	3-16	4	3-15	ND	ND	1	2.0
4A9κ	4-34	ARGKRVRTVWGRAIPASMASAFHF	23	2-2	3	3-20	QQHGTSPWT	10	1	3.8
4B9κ	4-34	ARGRRAGFRGERNFIAAVVASHYFDS	26	2-21	4	3-20	QQYGSSPET	9	2	1.9
4C1κ	3-30	AREFPWGSLDS	11	3-16	4	3-20	QQYGNSPSFWLT	12	4	3.9
4F8κ	3-23	ATNGYDNSGYFYPYYFHY	18	3-22	4	1-33	QQHDRLPIT	9	5	3.9
4F9κ	3-9	ARDIDPSYFYDRSGNAPFDY	20	3-22	4	3-20	QQYGSSPRT	9	1	4.0
4G1κ	4-34	ARGRRRIPPLASPRFQSQKKFYMDV	25	2-2	6	3-20	QQYGSSVWT	9	1	3.0
N-8B3κ	4-34	ARARAVRTAFGLPRRERKAEERRNWFDP	28	3-3	5	3-20	QQYGTSPYT	9	2	2.9
N-8C6κ	4-34	ARGRRAGYRGERNFIAAVIAAHYFDF	26	2-21	4	3-20	QQYGTSPET	9	2	2.4
N-8C10κ	4-30-4	ARGPHYADSSRGVFDV	16	3-22	3	1-5	QQCYSYPLT	9	4	1.4
N-8D3κ	4-34	VRGRRRIPPLASPRIKSQKKFYMDV	25	2-2	6	3-20	QQYGTSFWT	9	1	3.5
N-8D4κ	4-34	ARGRRAGFRGERTFIAAVVAAHYFDY	26	2-15	4	3-20	QQYGISPET	9	2	3.1
N-8D5κ	3-11	ARNMGKYDL	9	3-3	1	3-20	QHYDGSPIT	9	3	2.9
N-8E3κ	4-34	ARGRLIRSPFGQQRVPRQKSNWFDP	25	6-13	5	3-15	QQYNNWPPWT	10	1	2.5
N-8E6κ	3-30-3	ARDVRWLQLSY	11	5-24	4	1-13	QQYNGFPLT	9	4	2.6
N-8E10κ	1-18	ARGLLLYVGYYFDH	14	3-10	4	1D-13	QQFNYYPLT	9	4	3.4
N-8G1κ	3-49	AGGAEADSLIKYRYYYFYMDL	21	2-21	6	2-28	MQALQTPRT	9	2	2.7
N-8G3κ	4-34	ARGRRAGFRGERNFIAAVVASHYFDS	26	2-21	4	3-20	QQYGSSPET	9	2	1.8
N-8B12λ	4-34	ARGRRLRSAFDRSYNKRFTNYFDP	24	5-12	5	2-14	CSYRSSPTLYV	11	1	4.6
N-8C12λ	4-34	ARVPLPCSGGKCYLARVNRAADP	23	2-15	5	1-40	QSYDNSLSGYI	11	2	2.7
N-8E8λ	4-34	ARAFMPTSYLWGSHRRPPVDAFDI	24	3-16	3	2-23	CSHAGLNT	8	2	2.0
N-8F2λ	4-34	ARGLRQKSRMYYSASGSSYYFYMDV	25	3-10	6	1-51	GTWDSSLNVVV	11	2	2.4
N-8G2λ	5-51	ARRTSRPSSLNWGYRSTYAFDF	22	7-27	3	2-11	HSYAGTYEV	9	1	2.7

**Figure 8:**
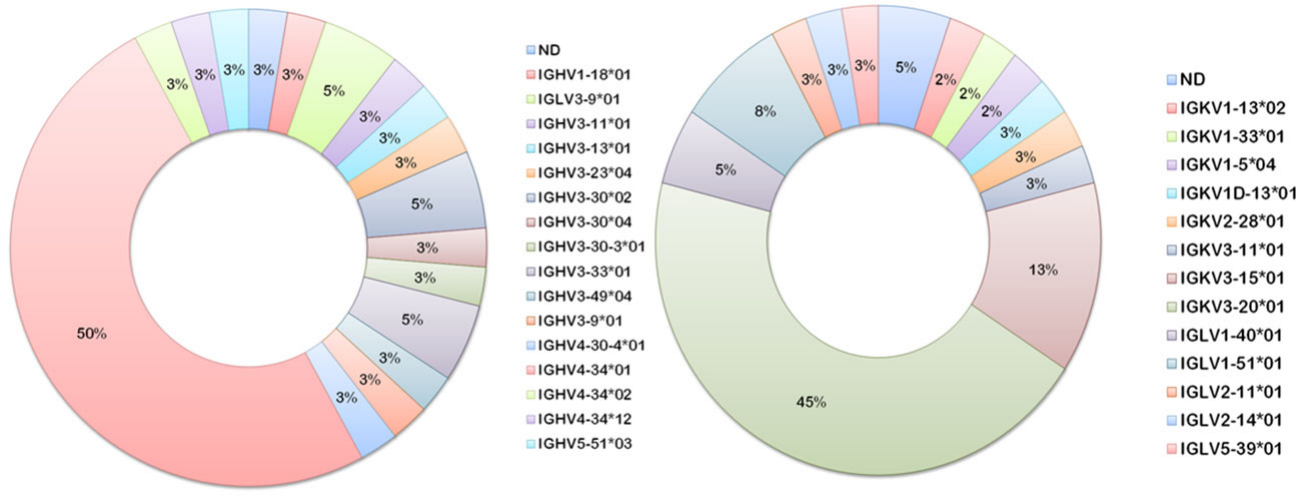
Ig V gene sequence usage in meningococcal vaccine antigen-specific hmAbs. Frequencies of usage of shown Ig gene sequence families for heavy (left donut) and light (right donut) chains of meningococcal protein-specific hmAbs.

Heavier usage of the IGHV4-34 gene was prominent amongst vaccinee-derived anti-meningococcal hmAbs cloned in this study (50%; 19/38, **Figure 8**). Similarly, the most used light chain V gene sequence was of the IGκV3-20 family (45%; 17/38, **Figure 8**). IgG hmAbs with this pairing (13/38), however, did not exhibit clonality in the diversity (D) gene regions, suggesting significant antigen-driven selection (**Table 2**).

**Figure 9:**
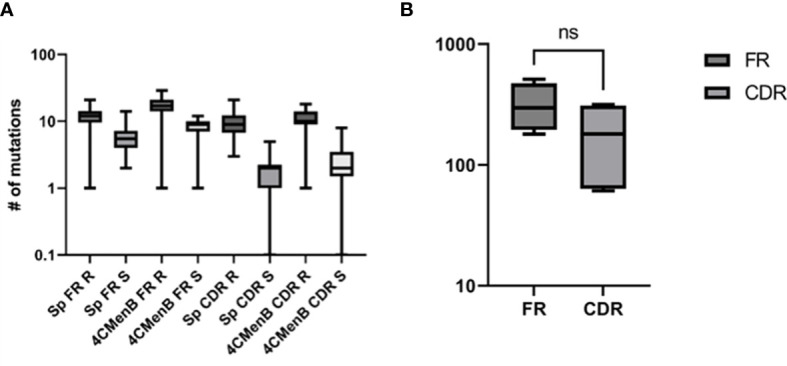
Characterisation of the sequence quality and length of V gene segments of cloned vaccine-specific hmAbs. **(A)** Replacement mutations were significantly more prominent than silent mutations across the different V gene regions. **(B)** A statistically non-significant ~1.7-fold difference was observed between frequencies of mutations in FR versus CDR. ns, non-significant.

The heterogeneity of vaccine antigen-specific hmAbs (sequence quality and length) driven by somatic hypermutation in rearranged VDJ genes was more pronounced in the heavy and light chain framework (FR) and complementarity-determining (CDR) regions. When compared to their respective germline sequences, more somatic mutations were enumerated in the structural stability-determining FR overall, but this difference in mutation frequency between FR (n = 1285) and CDR (n = 739) was not significant (p = 0.22). The replacement-to-silent mutation ratio (*d_N_*/*d_S_*) in both FR and CDR exceeded thresholds of 1.5 and 2.9, respectively, in 64.4% of hmAbs (mean *d_N_*/*d_S_* of 2.0 ± 0.11 and 4.5 ± 0.47, respectively) with replacement mutations observed to be significantly higher than silent mutations (p = 0.0134). Remarkably, CDR3 sequences in the VH gene segments of clonal hmAbs were not homogenous (**Figure 9** and **Supplementary Information Figures 4**, **5**).

## Discussion

In this study, enrichment of plasmablasts expressing vaccine-induced anti-pneumococcal and anti-meningococcal hmAbs using fluorescently labelled whole cell suspensions was successfully demonstrated. High antigen-specific hmAb cloning efficiency was observed with the mICA approach overall (~68%) compared with standard methods (~28%). The degree of enrichment obtained herein is largely comparable to other studies using single recombinant antigens: 42% with *Plasmodium falciparum* antigen GMZ2 (6), and 94% with HIV CN54gp140 protein (16). Some degree of variability in specific hmAb yield was observed, in line with reports in previously published literature. In this study, while enrichment of PCV13 and 4CMenB plasmablasts yielded high percentages of specific hmAbs (77% and 88%, respectively), standard methods also yielded a moderately high yield, but with the 4CMenB sample only (53%). In addition to the aforementioned studies (6, 16), Smith et al. achieved an average of 76% efficiency across four donors using non-enrichment methods (11) while another study that cloned PCV13 hmAbs using non-enrichment methods obtained a 51% yield (18 out of 35 hmAbs (26)). Other studies have reported variable efficiencies when cloning hmAbs specific to tetanus toxoid (23/56; 41%) and HIV-gp120 antigen (18/257; 7%) (27), and *Mycobacterium tuberculosis* (40%) (28), suggesting that other factors including timing of sample collection and individual differences in immune response magnitude (quantity and quality) are contributory to hmAb yield and may explain the low fold-increase following enrichment obtained with the 4CMenB plasmablasts. This variability in immunogenicity of the vaccines (either in the host response or antigen structure spheres) could also explain the total absence of hmAbs specific for the serotype 19F capsule, despite being part of the cell suspension probe. This inability to produce a panel of hmAbs composed of representatives of all PCV13 or PPSV23 vaccines was also reported previously (11, 26), and may be due to the aforementioned factors.

The presence of anti-pneumococcal and anti-meningococcal antibodies in pre-vaccination plasma is not uncommon, even expected, given the frequency of asymptomatic colonisation of the nasopharynx exhibited by both pathobionts, also known as carriage, in the human population and the consequent immune response (serum IgG included) to carriage (29, 30). It is reasonable to expect that enhanced proliferation of CD19+ cells expressing these carriage-induced anti-capsular and anti-protein antibodies would occur following vaccination thereby allowing for our mICA approach to enable isolation of both antigen targets. Indeed, it is believed that a proportion of the anti-meningococcal hmAbs cloned in this study would target non-4CMenB antigens and include *Neisserial* lipooligosaccharide epitopes (31). No anti-pneumococcal protein hmAbs were isolated suggesting that antigen-driven selection occurred following vaccination leading to the specificity of plasmablasts for the PCV13 polysaccharides only. This view is supported by the high *d_N_*/*d_S_* ratios in the CDRs and FRs of the hmAbs cloned herein. In contrast, no meningococcal polysaccharide-specific hmAbs were isolated in this study. It is worthy to note that because capsular types Y and B are the dominant serogroups in the UK (32), it is reasonable to expect that a higher likelihood of isolating hmAbs specific to the dominant meningococcal polysaccharide(s) in circulation existed. While the poor immunogenicity of the serogroup B polysaccharide (33) and the dominant Ig isotype to the serogroup B capsule being IgM (34) could explain the non-isolation of serogroup B polysaccharide-specific IgG plasmablasts from 4CMenB vaccinees, it is more plausible to expect that the quality of isolated vaccine-induced hmAbs would be dependent on the components of the vaccines. And hence the isolation of only polysaccharide- and protein-specific hmAbs from the pneumococcal polysaccharide and meningococcal protein vaccines, respectively. This non-isolation of other cellular components not contained in the vaccines does not, however, exclude the possible induction of antibodies targeting non-vaccine components from memory B-derived origins in the healthy subjects. Assessments of the exact 4CMenB targets of the anti-meningococcal hmAbs cloned in this study are in progress.

Concerning differences in hmAb affinity and/or functional property dependent on isolation method, i.e., standard versus mICA enrichment, there is no evidence from this present study and previous studies that employed standard methods that suggests that hmAbs obtained from either method would differ in affinity and/or functional property. In previously published work from our laboratory, for example, it was shown that three SBA hmAbs were cloned using standard methods (22) while an anti-pneumococcal hmAb from mICA enrichment in this present study possesses opsonophagocytic activity. This suggests that the differences in the affinity/functional attributes of individual hmAbs can be explained by the differences in cognate epitope specificity and not enrichment method.

Inactivation is an important consideration in preparing whole cell suspensions, especially in safety-sensitive situations. In this present study, live sorting of highly pathogenic pneumococcal and meningococcal strains with FACS facilities was not possible. Previous studies reported that formaldehyde-treatment inactivated but retained the structural integrity of whole cells to a degree that still allowed for display of surface epitopes that retained binding to: neutrophil extracellular traps *via* type IV pili (35); and, monoclonal and polyclonal anti-polysaccharide antibodies (36). In addition, formaldehyde-treatment was also employed in a previous study that successfully induced a targeted and homogenous IgG response to the pneumococcal polysaccharide for antibody biochemistry purposes (37). Hence, formaldehyde-treatment was chosen as an inactivation method in this present study. While formaldehyde is an effective inactivation agent, it can cross-link proteins, DNA, and polysaccharides (38, 39). This cross-linking attribute has identified the importance of conformational antibodies in the immune response to meningococci (40). In this present study, formaldehyde cross-linking may have altered protein and/or polysaccharide epitopes such that they were not recognisable by cloned hmAbs, and is a possible limitation of the approach. Alternative inactivation methods for when live sorting is not possible include UV radiation (at least, for specific strains such as the hypervirulent meningococcal ST-11 clonal complex; 41), antibiotic treatment, or heat killing. For UV treatment, however, investigations into the dose, concentration, temperature and time of treatment would be required to determine the extent of bacterial cellular disruption. Heating meningococci for 60 minutes at 50^0^C or 60^0^C was found to be more effective than formaldehyde-treatment when using polyclonal antibodies in agglutination, co-agglutination, latex kit testing, and serotyping by dot-blot ELISA (42), but cellular intactness was not reported. Nevertheless, hmAbs cloned *via* mICA, and using formaldehyde-treated bacterial suspensions in this present study, recognized cognate epitopes on viable bacteria in functional assays, demonstrating that hmAb cognate epitope integrity was maintained following formaldehyde treatment.

In summary, given the ongoing need for effective vaccines, the robust proliferation of antigen-specific plasmablasts in patients at early stages of convalescence can be harnessed for vaccine antigen discovery. The results presented in this study provide proof-of-principle and establish the usefulness of a bacterial whole-cell ICA in the enrichment of antigen-specific plasmablasts for enhanced hmAb cloning precision, thereby increasing the power of RV 2.0. It is expected that this approach will be especially valuable in studies utilising patient plasmablasts where restrictions on blood sample volume may exist (as in the case of paediatric patients) or in studies where the magnitude of the immune response (peak plasmablast timing) to the pathogen is yet to be described. While whole cells of both Gram-positive and Gram-negative bacteria have been used in this study as a representation of a complex antigenic probe, we envisage further modifications to this protocol (for example, use of lysine-reactive fluorescent dyes) that will enable the isolation of pathogen-specific plasmablasts using other complex antigen probes such as OMV preparations.

## Data availability statement

The sequence data presented in this study are deposited in the GenBank repository, accession numbers OR025766 - OR025877.

## Ethics statement

Ethical review and approval was not required for the study on human participants in accordance with the local legislation and institutional requirements. The patients/participants provided their written informed consent to participate in this study.

## Author contributions

Conceptualization: FB, PL, CP, PM and RS. Investigation and experimental execution: FB, SS, YG, CG and RP. Writing – Original Draft: FB. Writing – Reviewing & Editing: All authors.
