# Supplementary material for: Increasing human monoclonal antibody cloning efficiency with a whole-cell modified immunoglobulin-capture assay (mICA)
Source: Front Immunol. 2023 Jun 2;14:1184510. doi: 10.3389/fimmu.2023.1184510 (PMC10272928; doi:10.3389/fimmu.2023.1184510)
Supplement: Supplementary file 1 [file DataSheet_1.pdf]

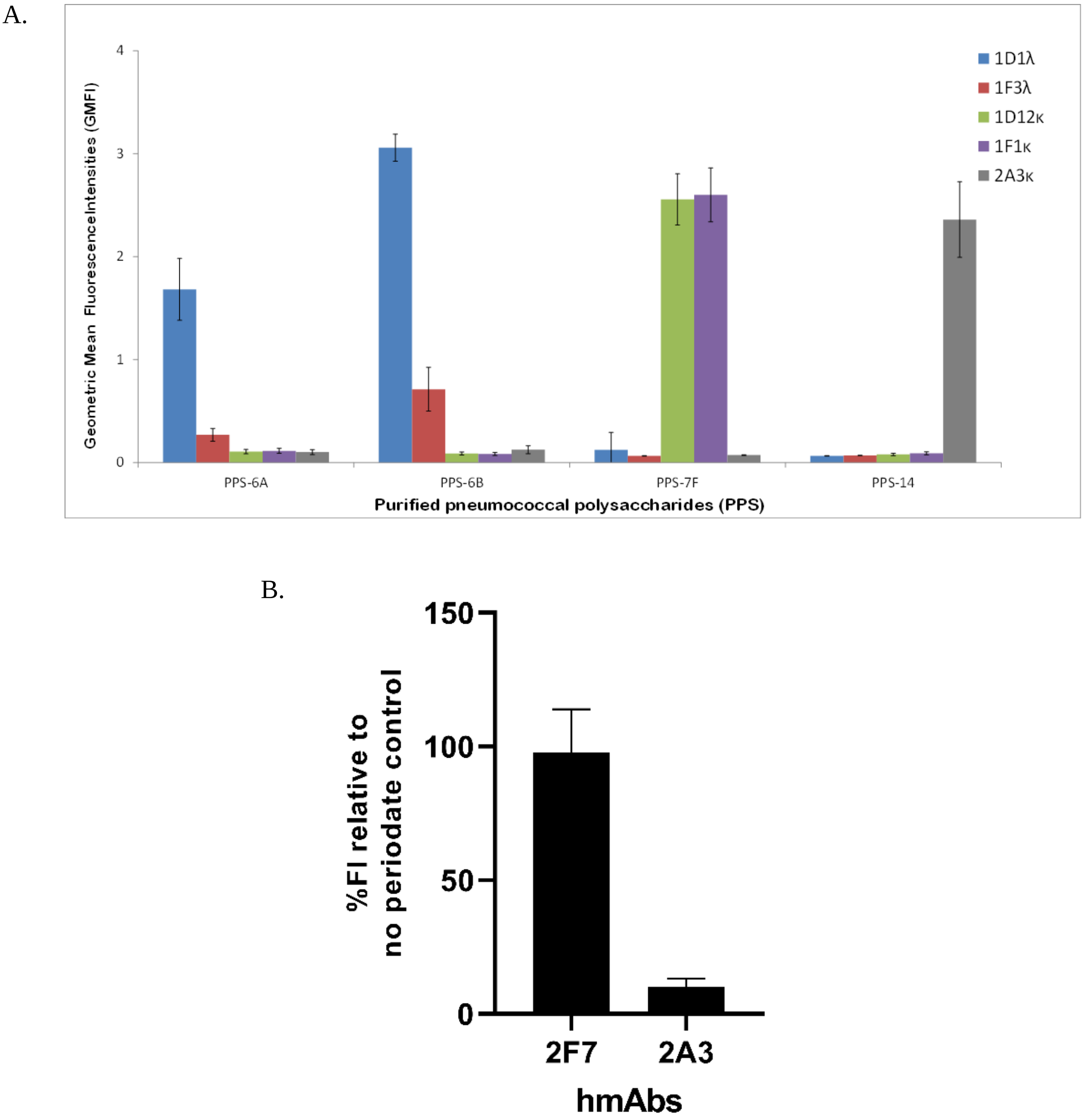

Figure 1: Geometric mean fluorescence intensity (GMFI) of selected antibodies in ELISAs versus PPS of serotypes 6A, 6B, 7F and 14 (panel A) and the ELISA-based sodium periodate assay (panel B) were obtained from 3 independent experiments; error bars represent standard error of the mean from nine data points (3x technical replicates in 3x independent experiments/biological replicates).

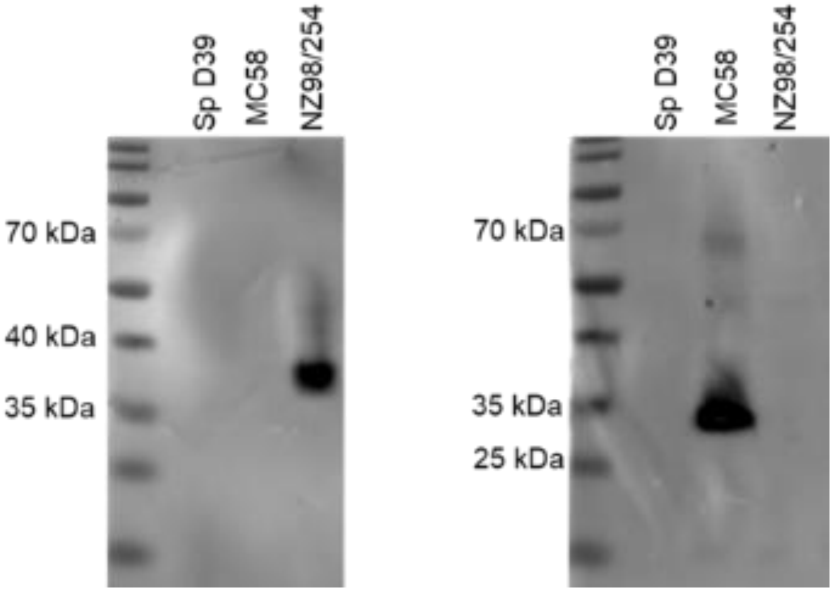

Figure 2: Reactivity of hmAb 3G3 (from mICA, left) and 8D5 (from non-mICA, right) with a strain NZ98/254 OMV composite antigen and factor-H binding protein, FHbp, respectively.

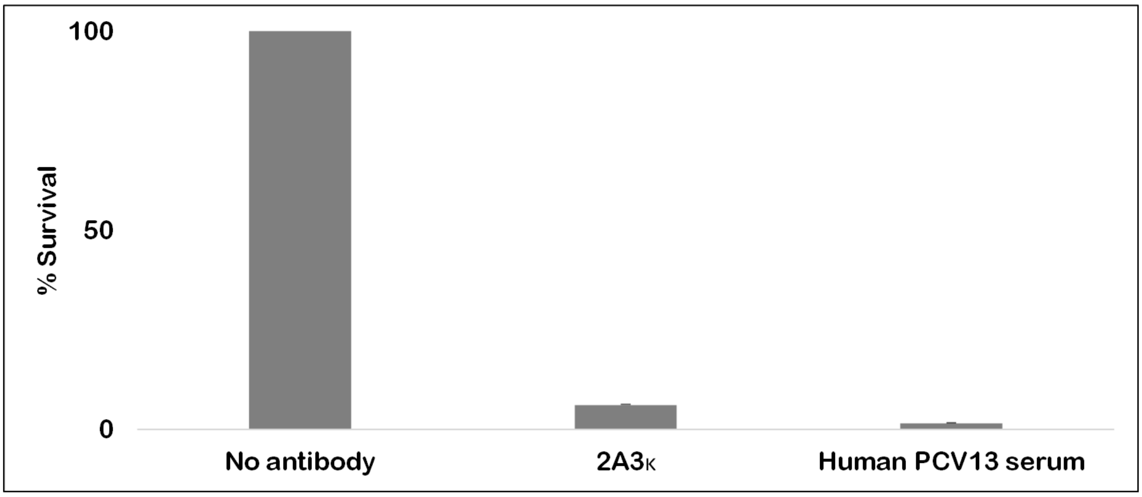

Figure 3: Ability of serotype 14-specific hmAb, 2A3κ, to mediate uptake and killing of the homologous strain M117-14. Percentage OPKA of 2A3κ was expressed relative to reduction in CFU levels of a no-hmAb control following a 60-minute incubation in presence of exogenous human complement. Human immune plasma from pooled vaccinee samples (PPSV23 plus PCV13) served as positive control.

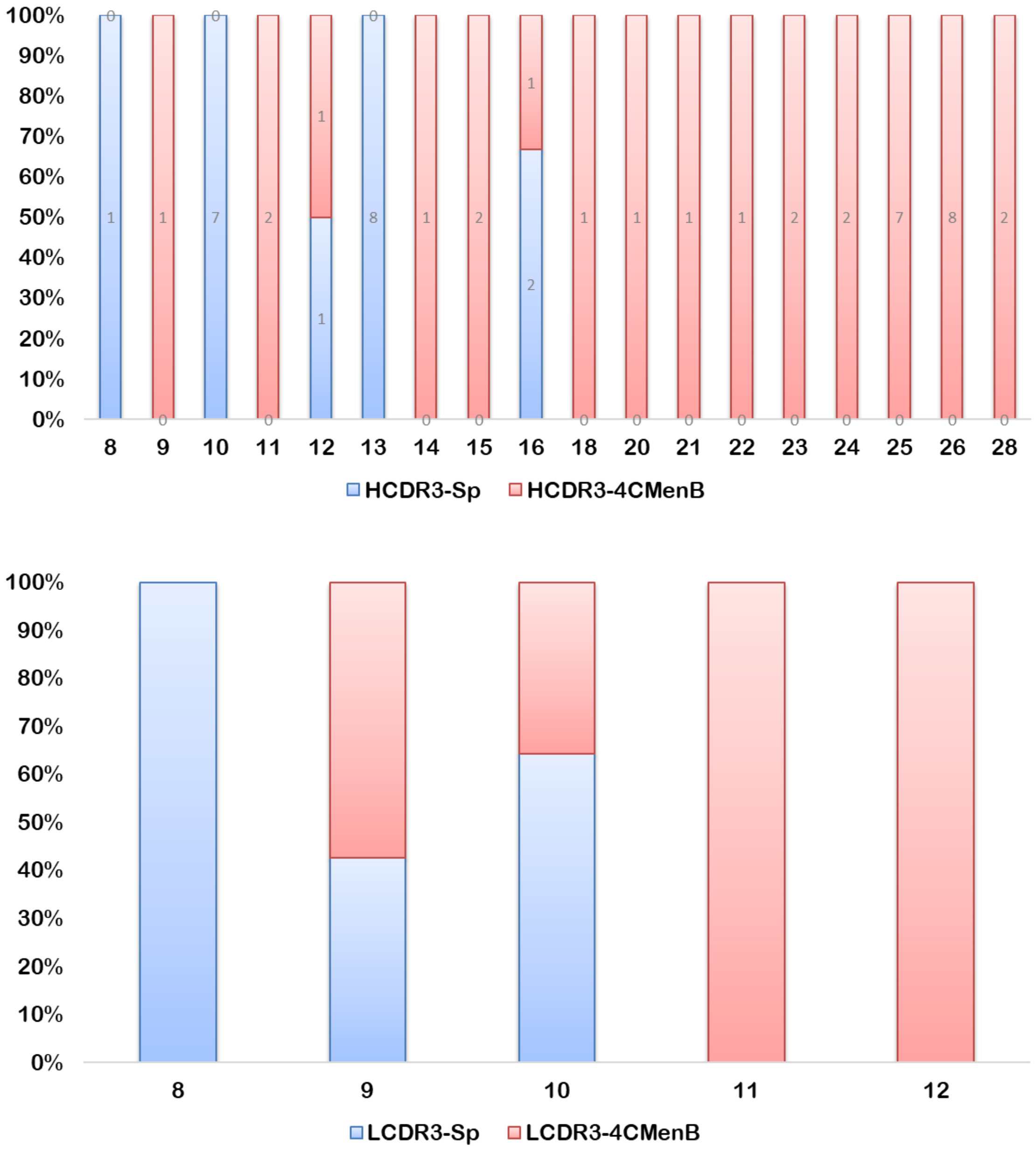

Figure 4: Lengths of the CDR3 regions of heavy **(A)** and light **(B)** chains of antigen-specific hmAbs; higher length variability was present amongst anti-meningococcal vaccine-induced hmAbs. **(E)** Representative example of hmAb heterogeneity evidenced by sequence alignment of CDR3 regions of clonally related anti-meningococcal (IGHV4-34:IGκV3-20 pairing) hmAbs.

```
3C1κ     --------------------VRGRRRIP-PLASPRIQSQRRFYMDV   25
3E10κ    -------------ARGRRAGYRGERNFFAPMVA-------AHYFDS   26
3F11κ    -------------ARGRRAGFRGERNFIAAVVA-------AHYFDY   26
3G1κ     ARARAVLTAFGSPRRERKAGE--RRNWFDP----------------   28
3G2κ     -------------ARGRRAGFRGERNFIAAVVA-------SHYFDS   26
3G3κ     -------------SRGRRAGFRGERNFFAAMVA-------SHYFDS   26
4A9κ     -------------ARGKRVRTVWGRAIPASMAS-------AFHF--   24
4B9κ     -------------ARGRRAGFRGERNFIAAVVA-------SHYFDS   26
4G1κ     --------------------ARGRRRIP-PLASPRFQSQKKFYMDV   25
8C6κ     -------------ARGRRAGYRGERNFIAAVIA-------AHYFDF   26
8D3κ     --------------------VRGRRRIP-PLASPRIKSQKKFYMDV   25
8D4κ     -------------ARGRRAGFRGERTFIAAVVA-------AHYFDY   26
8G3κ     -------------ARGRRAGFRGERNFIAAVVA-------SHYFDS   26
                                 *
```

Figure 5: Representative example of hmAb heterogeneity evidenced by sequence alignment of CDR3 regions of clonally related anti-meningococcal (IGHV4-34:IGκV3-20 pairing) hmAbs.

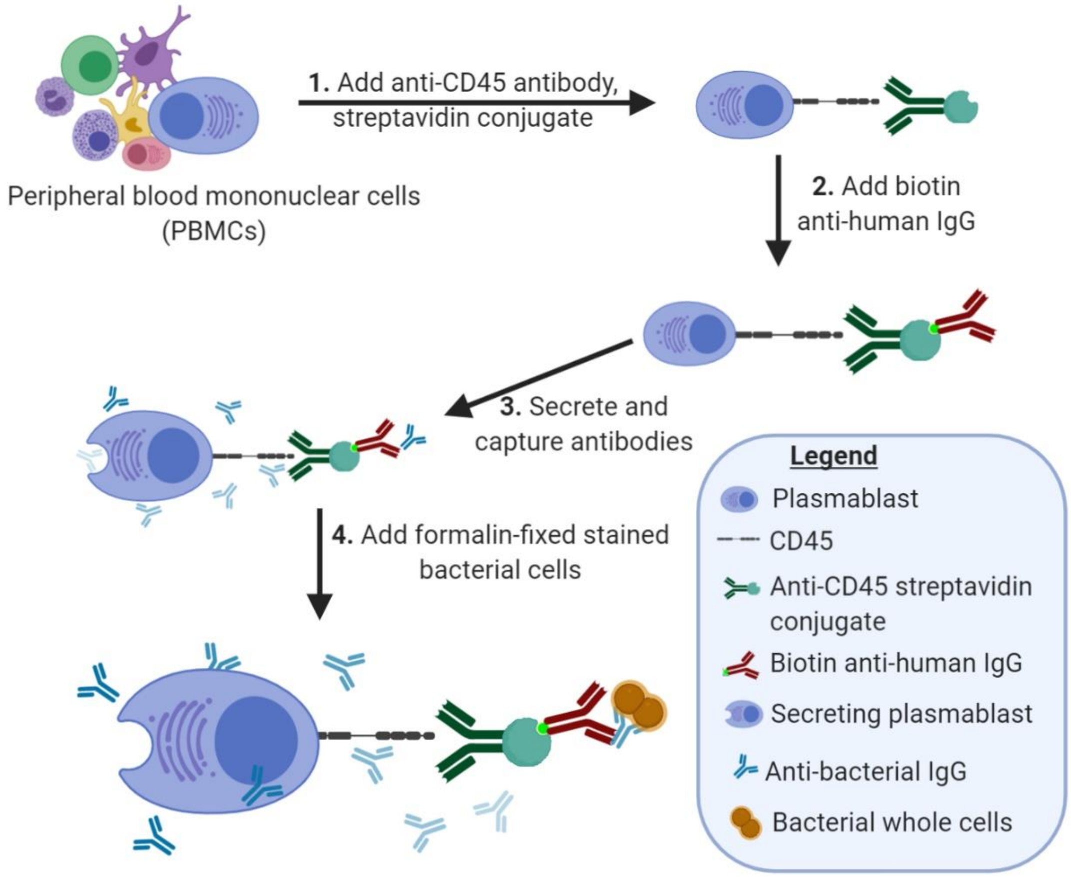

Figure 6: Schematic representation of the mICA protocol. Reproduced with permission from Springer Nature (Siris *et al.*, 2021).
